# Genome-wide association study for the primary feather color trait in a native Chinese duck

**DOI:** 10.3389/fgene.2023.1065033

**Published:** 2023-03-03

**Authors:** Yanfa Sun, Qiong Wu, Rulong Lin, Hongping Chen, Min Zhang, Bingbing Jiang, Yaru Wang, Pengfei Xue, Qiuyun Gan, Yue Shen, Feifan Chen, Jiantao Liu, Chenxin Zhou, Shishi Lan, Haozhe Pan, Fan Deng, Wen Yue, Lizhi Lu, Xiaobing Jiang, Yan Li

**Affiliations:** ^1^ College of Life Sciences, Longyan University, Longyan, Fujian, China; ^2^ Fujian Provincial Key Laboratory for the Prevention and Control of Animal Infectious Diseases and Biotechnology, Longyan, Fujian, China; ^3^ Fujian Provincial Universities Key Laboratory of Preventive Veterinary Medicine and Biotechnology (Longyan University), Longyan, Fujian, China; ^4^ Longyan Shan-ma Duck Original Breeding Farm, Agricultural Bureau of Xinluo District, Longyan, Fujian, China; ^5^ Institute of Animal Science and Veterinary, Zhejiang Academy of Agricultural Sciences, Hangzhou, China; ^6^ Fujian Provincial Animal Husbandry Headquarters, Fuzhou, Fujian, China

**Keywords:** Longyan Shan-ma duck, primary feather color trait, GWAS, candidate gene, *STARD9*

## Abstract

**Background:** To reveal candidate genes and the molecular genetic mechanism underlying primary feather color trait in ducks, a genome-wide association study (GWAS) for the primary feather color trait was performed based on the genotyping-by-sequencing (GBS) technology for a native Chinese female duck, Longyan Shan-ma ducks.

**Methods:** Blood genomic DNA from 314 female Longyan Shan-ma duck were genotyped using GBS technology. A GWAS for the primary feather color trait with genome variations was performed using an univariate linear mixed model based on all SNPs in autosomes.

**Results:** Seven genome-wide significant single nucleotide polymorphisms (SNPs, Bonferroni-adjusted *p*-value <8.03 × 10^−7^) within the introns of the genes *STARD9*, *ZNF106*, *SLC7A5*, and *BANP* genes were associated with the primary feather color trait. Twenty-two genome-wide suggestive SNPs (Bonferroni-adjusted *p*-value <1.61 × 10^−5^) of 17 genes (besides *ZNF106* and *SLC7A5*) were also identified. Seven SNPs were located at one 0.22 Mb region (38.65–38.87 Mb) on chromosome 5, and six SNPs were located at one 0.31 Mb region (19.53–19.84 Mb) on chromosome 11. The functions of *STARD9*, *SLC7A*5, *BANP*, *LOC101798015*, and *IPMK* were involved pigmentation and follicle development, especially, *STARD9* upregulated expression in black feather (haplotype-CCCC) bulb tissue compared with in pockmarked feather (haplotype-TGTT) bulb tissue, implicating these genes as candidate genes for primary feather color trait.

**Conclusion:** The preliminarily findings suggested candidate genes and regions, and the genetic basis of primary feather color trait in a female duck.

## Introduction

Feathers are one of the most peculiar derivatives of avian skin (Alibardi and Toni, 2008). In ducks, feather color is an important economic trait and is considered a breed characteristic (Lin et al., 2022). As a complex trait, feather color of ducks is controlled by multiple genes located at different loci. Interactions of these loci produce abundant feather phenotypes, such as recessive white and extended black (Li et al., 2012). The genetic basis underlying feather color in ducks has been extensively studied (Li et al., 2015; Sultana et al., 2018; Zhou et al., 2018; Yang et al., 2019; Xi et al., 2021). Li et al. found that two single nucleotide polymorphisms (SNPs; c.940G>A and c.995G>A) of the endothelin receptor B2 gene (*EDNRB2*) were associated with spot feather pattern in ducks using a three-generation intercross between white Kaiya and white Liancheng ducks (Li et al., 2015). Another study using five Asian duck varieties showed that two synonymous SNPs (c.114T>G and c.147T>C) and a 14-bp insertion-deletion (indel) (GCTGCAAAC AGATG) in intron 7 of the microphthalmia-associated transcription factor gene (*MITF*), one non-synonymous SNP [c.938A>G (p.His313Arg)] and a synonymous SNP (c.753A>G) of the dopachrome tautomerase gene (*DCT*) were significantly associated with the black and white color feather trait (Sultana et al., 2018). Yang et al. found that six SNPs of *MITF* were associated with different feather color pattern of ducks (Yang et al., 2019). By analyses of sequencing data of 106 Pekin ducks, Zhou et al. confirmed that an approximately 6.6-kb insertion led to a splicing change in *MITF*. The change, which does not exist in other duck breeds or mallards, leads to white down feathers in Pekin ducks (Zhou et al., 2018). Using the intercross population from a cross between the GF2 strain and Jianchang duck with 225 duck, Xi et al. demonstrated by a genome-wide association study (GWAS) that two SNPs (Chr4: 10,180,939 T > C and Chr4: 10,190,671A > T) of the endothelin B receptor-like gene (*EDNRB2*) explained most of the duck body surface spot size (Xi et al., 2021). In the foregoing studies, candidate genes and GWAS methods were used to identify the genes controlling feather color in different populations and breeds of ducks. However, the genetic basis of local feather variation in different breeds of ducks remains unclear. GWAS provide an opportunity to comprehensively and systematically uncover the genetic underlying black feather trait in duck (Guo et al., 2022).

Ducks are valuable sources of meat, eggs, and feathers. They are one of the most economically important waterfowl (Lin et al., 2015). There are more than 20 laying-type duck breeds in China. The Shan-ma duck, one of the main indigenous egg laying duck breeds, is commercially valuable, with 250–300 million ducks raised annually, representing approximately half the laying ducks raised in China. The Shan-ma duck originated in Longmen town of Longyan city, in China. The Ministry of Agriculture and Rural Affairs of China officially approved the registration and protection of geographical indication of agricultural products for “Longyan Shan-ma duck” in January 2017. Most female Longyan Shan-ma ducks are covered with pockmarked feathers in the primary feather, with only one or several black feathers in the primary feather.

Herein, a GWAS for primary feather color trait was performed using the genotyping-by-sequencing (GBS) strategy to identify candidate genes and explore the genetics basis of the primary feather color trait in Longyan Shan-ma ducks.

## Materials and methods

### Birds and phenotype

The fourth-generation high yield line Longyan Shan-ma female ducks were raised at the Longyan Shan-ma Duck Original Breeding Farm under the same environmental and nutritional conditions. These ducks were incubated at the same time. These ducks in starting and growing periods were raised in a large cage. The laying period was in individual cages. Three hundred and fourteen female ducks were used in this study. The number of pockmarked or black feathers in primary feathers on one side wing (i.e., the feather color traits) were measured at 400-day-of-age. The feathers included pockmarked feathers, one black feather and multiple black feathers. At the same time, feather bulb tissues of ducks were obtained and preserved in RNAfixer sample preservation solution (Bioteke, Beijing, China) at −80°C until used.

### Genotyping, annotation, quality control, and imputation

In 504-day-old ducks, blood was sampled from the brachial vein using citrate-based anticoagulant syringes, snap-frozen in liquid nitrogen, then stored at −80°C until used. Genomic DNA (gDNA) was isolated from blood samples using the traditional phenol-chloroform method. The quality and concentration of gDNA met the specific requirements for library construction of double-digest GBS, as previously suggested (Wang et al., 2017). A total of 314 qualified gDNA samples were genotyped using GBS. Libraries were sequenced on an Illumina Novaseq™ platform (PE150). Clean data (clean reads) were obtained from raw data by removing adapters, poly-N reads and low-quality reads. Clean data was aligned to the duck reference genome (PK-2015) (Huang et al., 2013) using Burrows-Wheeler Aligner software (Li and Durbin, 2009). The raw SNP sets were called by SAMtools software (Li et al., 2009), with the parameters as “-q 1 -C 50 -t AD, DP -m 2 -F 0.002”. SNPs were further annotated using ANNOVAR (Wang et al., 2010).

Using Plink software, version 1.9, samples and SNPs were excluded for failing to meet one or more of the following conditions: sample call rate <80%, SNP call rate <90%, minor allele frequency (MAF) < 5%, Hardy-Weinberg equilibrium test *p* < 10^−6^, and SNPs on the Z chromosome. After quality control, missing genotypes were imputed using Beagle 5.4 (version: 22Jul22.46e) (Browning et al., 2018) with the default parameter.

### Independent SNPs and population structure

Independent SNPs were acquired using Plink 1.9 software through all autosomal SNPs pruned using the indep-pairwise option, with a window size of 25 SNPs, step of five SNPs, and threshold *r* ^2^ = 0.2 as previously suggested (Liu et al., 2013; Sun et al., 2013). According to these independent SNPs, the top ten PCs of each sample were calculated using Plink 1.9 software. The population structure plot was drawn using principal component 1 as the *X*-axis and principal component 2 as the *Y*-axis. To assess whether the population of ducks used in this study were stratified or not, clustering analysis were performed using Plink 1.90 software, and PC significance test was conducted using the EIGENSTRAT (version 6.1.4) software (Price et al., 2006).

### Association analysis

SNPs-trait association analysis was performed using an univariate linear mixed model LMM based on all SNPs in autosomes using the GEMMA software, v0.98.4 (Zhou and Stephens, 2012). The centered kinship matrix (K) was calculated based on all SNPs in autosomes using GEMMA v0.98.4 software. The statistical model was calculated as:

$$Y_{ijlm}=\mu_{i}+{PCs}_{j}+{K_{l}+G}_{m}+e_{ijlm}$$

where $Y_{ijlm}$ are phenotypic values, $\mu_{i}$ is the common mean, ${PCs}_{j}$ are the effect of the PC, $K_{l}$ is the kinship matrix, $G_{k}$ is the effect of the SNP and lm is the random residual. The phenotypic values are treated as numerical values. When the duck population used in this study displayed stratification, the corresponding number of PCs were fit in the model as covariates, or were excluded from the model. The significance of the associations was determined with a Wald test.

A genome-wide significance threshold (0.05 divided by the number of markers) and suggestive significance threshold (1 divide by the number of markers) were determined using a Bonferroni correction to reduce false positive probability. The Quantile-quantile and Manhattan plots for the GWAS results were generated with “CMplot” package using R software (v4.1.3).

### Function enrichment analyses of identified associated genes

GO term and KEGG pathway enrichment analyses for the GWAS identified associated genes of primary feather color trait in ducks were performed using KOBAS-i web-based software (Bu et al., 2021).

### Q-PCR for *STARD9* gene

Top three significantly associated SNPs were in the *STARD9* gene, therefore *STARD9* gene was chose for further validation. Feather bulbs tissues from three ducks with black feather bulbs (haplotype-TGTT) and three ducks with pockmarked bulbs (haplotype-CCCC) were obtained, and stored at RNA stock solution RNAfixer (RA110) (Biomed Gene Technology, Co., Ltd., Beijing, China). Total RNA from feather bulbs tissue was extracted using standard TRIZOL RNA extraction protocol. High quality total RNA of each sample (A260/A280 > 1.90, concentration >500 ng/μL) was used for GBS library construction.

Synthesis of first-strand cDNA from 1,000 ng total RNA was performed using the TUREscript 1st Stand cDNA SYNTHESIS Kit (Aidlab Biotechnologies, Beijing, China). The expressions of mRNAs were measured using SYBR^®^ Green Master Mix (DBI Bioscience, Shanghai, China) and performed using a qTOWER 2.0/2.2 apparatus (Analytik Jena, Jena, Germany). The primers were designed using Beacon Designer 8.14 (PREMIER Biosoft, Palo Alto, CA, United States). The primers of gene *STARD9* were F-AAAGCCTGCCAAATTGAGTC and R-AAAGGTATGACAGGTCCCAAA (NCBI reference sequence XM_027458585.2). Beta (β)-actin was used as internal reference gene. The β-actin primers were F-GTACTCTGTCTGGATTGGA, and R-ATCATACTCCTGCTTGCT.

Each amplification was carried out in a volume of 20 μL, containing 5 μL of 2×SYBR^®^ Green Master Mix, 100 ng cDNA of each candidate gene or internal reference gene and 0.5 μL of each primer (10 μmol), with distilled deionized water added to a total volume of 20 μL. The housekeeping β-actin gene was used as internal reference gene for Q-PCR. The following reaction conditions were used: 95°C for 3 min, followed by 40 amplification cycles of 95°C for 10 s, 58°C for 30 s, melt curve analysis 60–95°C and +1°C/cycle, with a holding time of 4 s. The comparative CT method (ΔΔCT) was used to determine fold-changes in gene expression level (Livak and Schmittgen, 2001). Gene expression levels between pockmarked and black feather bulb tissues in ducks were performed with independent-sample *t*-test using SPSS version 19.0 (IBM, Armonk, NY, United States). All results are expressed as mean ± standard deviation. Differences between two groups were tested using Student’s *t*-test statistic at a level of significance of *p* < 0.05.

## Results

### Feather color trait

The pockmarked (A) and black (B) feather traits of Longyan Shan-ma ducks are shown in Supplementary Figure S1. According to the number of pockmarked or black feathers in primary feathers on one side wing, the feather color traits were recorded as pockmarked feathers (*n* = 261), one black feather (*n* = 20), and two or more black feathers (*n* = 9). In all, 290 individuals with genotype and phenotype information were included in the analysis.

### SNP markers

A total of 99,800 SNPs in autosomes were acquired using GBS technology. After quality control, 11 samples and 37,534 SNPs were excluded, 303 ducks and 62,266 SNPs remained for further analysis. The remaining SNPs were distributed among 29 chromosomes and approximately 63.41 SNPs per Mb (Supplementary Table S1).

### Independent SNPs and population structure

**FIGURE 1:**
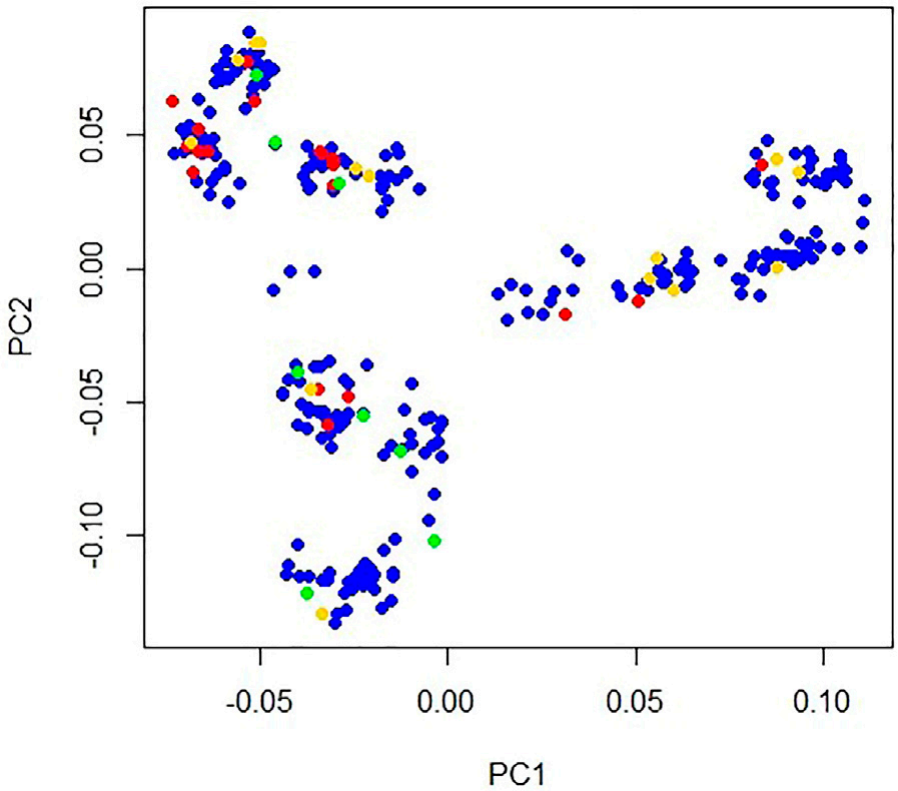
Population structure plot of Longyan Shan-ma duck. *X*-axis represents principal component 1 and *Y*-axis represents principal component 2. Blue dot indicates pockmarked feathers (*n* = 261), red dot indicates one black feather (*n* = 20), green dot indicates multiple black feathers (*n* = 9), and gold dot indicates missing phenotype (*n* = 13) on one side wing.

A total of 10,210 independent SNPs (Supplementary Table S1) were obtained with *r* ^2^ = 0.2 multidimensional scaling (MDS) analysis of these 62,266 SNPs on 29 autosomes using PLINK V1.9 software (Chang et al., 2015). Using the principal component command of this software, the top ten principal components (PCs) were obtained using these independent SNPs. A population structure plot was drawn using the first two PCs (Figure 1). Top two PCs explained 16.57% and 12.49% population structure variance, respectively. Clustering analysis in Plink 1.90 software revealed a cluster the population of ducks in this study. A significance test of PCs performed using EIGENSTRAT (version 6.1.4) software (Price et al., 2006) indicated that the PCs were not significant (*p* = 0.15) (Supplementary Table S2). The findings indicated that the population of the ducks used in this study was not stratified. PCs were removed as covariates in the association analysis.

### GWAS results

**FIGURE 2:**
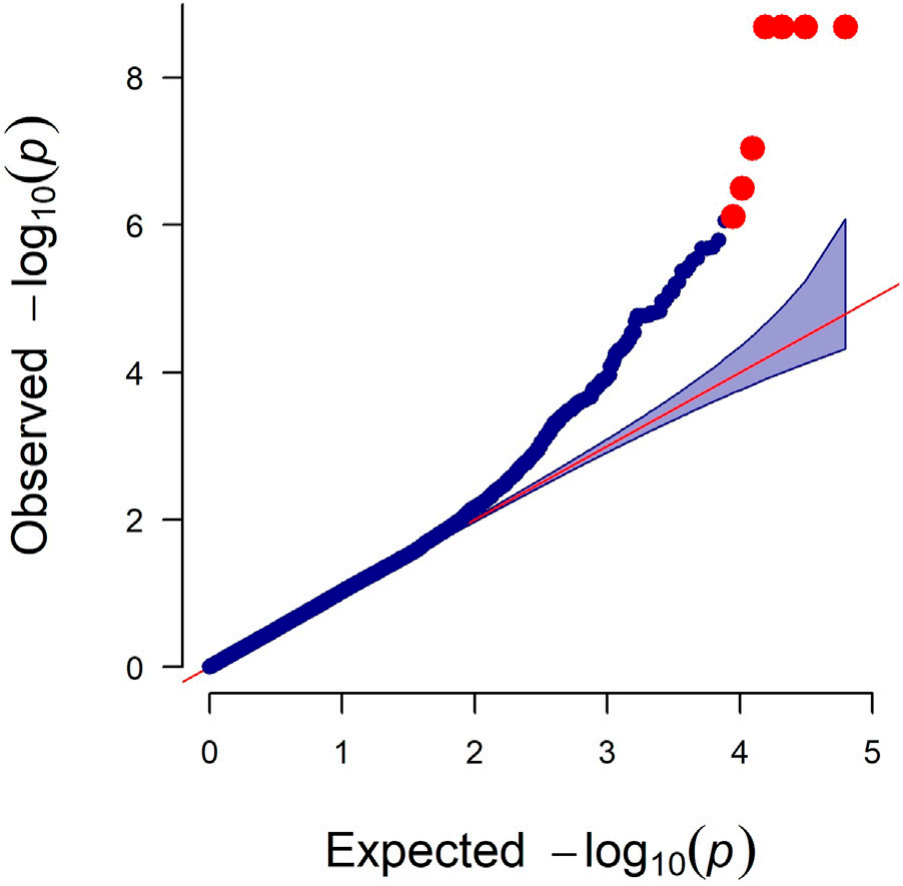
Quantile-quantile plot of genome-wide study association study for the primary feather color trait in ducks. The *x*-axis indicates the expected −log 10 *p*-values. The *y*-axis indicates the observed −log10 *p*-values.

**FIGURE 3:**
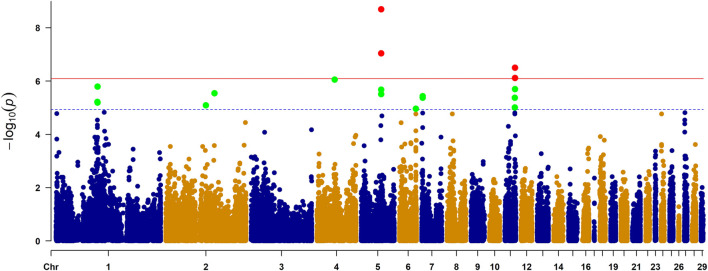
Manhattan plot of genome-wide study association study for the primary feather color trait in ducks. The *X*-axis represents the genomic position of corresponding SNP and the *Y*-axis represents −log_10_-tansformed *p*-value of the associations. Each dot represents each SNP. The red line represents *p*-value = 8.03 × 10^−7^ (0.05/62,266) for the Bonferroni adjusted genome-wide significance threshold, and blue line represents *p*-value = 1.61 × 10^−5^ (1/62,266) for the Bonferroni adjusted genome-wide suggestive significance threshold. Four genes reaching genome-wide significance were indicated in Manhattan plot, including *STARD9*, *ZNF106*, *SLC7A5*, and *BANP*.

As observed in a quantile-quantile plot (Figure 2), the LMM containing the kinship matrix effectively reduced the false associations due to genetic relatedness (Sul et al., 2018). Seven genome-wide significant and 22 suggestive associations (27 SNPs) for primary feather color trait were identified based on Bonferroni adjusted genome-wide significance threshold (0.05/62,266, *p*-value = 8.03 × 10^−7^) and genome-wide suggestive significance threshold (1/62,266, *p*-value = 1.61 × 10^−5^) (Figure 3; Table 1; Supplementary Table S3). For these identified SNPs, higher allele (A1) frequencies were associated with increased the number of primary black feather in Longyan Shan-ma duck, odds ratio (OR) ≥ 1.20 (Table 1; Supplementary Table S3).

**TABLE 1:** SNPs with genome-wide significance associations for plumage color traits.

Chromosome	SNP	BP (PK-2015)^a^	A1/A2	A1-MAF^b^	OR (95% CI)^c^	*p*-value	Gene	Feature
5	chr5:38647451 C>T	38,647,451	T/C	0.067	1.47 (1.28 ± 1.69)	9.12 × 10^−8^	*ZNF106*	intron
5	chr5:38713690 C>T	38,713,690	T/C	0.071	1.52 (1.33 ± 1.74)	2.03 × 10^−9^	*STARD9*	intron
5	chr5:38743433 C>G	38,743,433	G/C	0.071	1.52 (1.33 ± 1.74)	2.03 × 10^−9^	*STARD9*	intron
5	chr5:38743534 C>T	38,743,534	T/C	0.071	1.52 (1.33 ± 1.74)	2.03 × 10^−9^	*STARD9*	intron
5	chr5:38743601 C>T	38,743,601	T/C	0.071	1.52 (1.33 ± 1.74)	2.03 × 10^−9^	*STARD9*	intron
11	chr11:19693957 G>A	19,693,957	A/G	0.095	1.34 (1.20 ± 1.50)	7.61 × 10^−7^	*SLC7A5*	intron
11	chr11:19840846 C>T	19,840,846	T/C	0.100	1.34 (1.20 ± 1.49)	3.16 × 10^−9^	*BANP*	intron

^a^ BP, physical position based on duck reference genome PK-2015 (https://www.ncbi.nlm.nih.gov/genome/2793?genome_assembly_id=426073).

^b^ MAF, minor allele frequency.

^c^ OR, odds ratio; 95% CI, 95% confidence interval.

For the seven genome-wide significant associations, four SNPs (chr5:38713690 C>T, chr5:38743433 C>G, chr5:38743534 C>T, and chr5:38743601 C>T) were located on chromosome 5 within the intron of the StAR related lipid transfer domain containing the StAR related lipid transfer domain containing 9 gene (*STARD9*) (Table 1). Of the remaining SNPs, one (chr5:38647451 C>T) was located on chromosome 5 within the intron of the zinc finger protein 106 (*ZNF106*), one (chr11:19693957 G>A) was located on chromosome 11 within the intron of the solute carrier family 7 member 5 (*SLC7A5*), and one (chr11:19840846 C>T) was located on chromosome 11 within the intron of the BTG3 associated nuclear protein gene (*BANP*) (Table 1).

For 22 suggestive associations, 22 SNPs were located on chromosomes 1, 2, 4, 5, 6,7, 11, and 27 within intron (14), intergenic region (5), exon (1), 5′ untranslated region (UTR5) (1), and 3′ untranslated region (UTR3) (1) of 17 genes, including cystathionine beta-synthase (*LOC101798015*), crystallin alpha A (*CRYAA*), salt inducible kinase 1 (*SIK1*), chloride intracellular channel 6 (*CLIC6*), PH domain and leucine rich repeat protein phosphatase 1 (*PHLPP1*), secretagogin, EF-hand calcium binding protein (*SCGN*), zinc finger protein 827 (*ZNF827*), *ZNF106*, tau tubulin kinase 2 (*TTBK2*), inositol polyphosphate multikinase (*IPMK*), LIM domain only 1 (*LMO1*), tripartite motif containing 66 (*TRIM66*), zinc finger CCHC-type containing 14 (*ZCCHC14*), kelch domain containing 4 (*KLHDC4*), *SLC7A5*, plexin A2 (*PLXNA2*), and calcium/calmodulin dependent protein kinase IG (*CAMK1G*) (Supplementary Table S3). One SNP (chr2:94385810 C>T) caused a non-synonymous mutation (S113N), changing serine to asparagine, exon five of the *SCGN* gene.

It should be emphasized that some identified genome-wide significant and/or suggestive significant SNPs are located in one region of the chromosome. On chromosome 5, eight SNPs located within an 0.22 Mb region (38.65–38.87 Mb) were associated with primary feather color trait (*p*-value ≤2.10 × 10^−6^), including four SNPs (chr5: 38713690 C>T, chr5: 38743433 C>G, chr5: 38743534 C>T, and chr5: 38743601 C>T) within the intron of gene *STARD9*, three SNPs (chr5: 38647398 G>T, chr5:38647451 C>T, and chr5: 38647483 A>G) within the intron of gene *ZNF106*, and one SNP (chr5:38866077 T>C) within the intron of gene *TTBK2*. On chrosomose 11, six SNPs were located within the 0.31 Mb region (19.53–19.84 Mb), including one SNP (chr11:19840846 C>T) within the intron of the gene *BANP*, two SNPs (chr11: 19695701 G>A and chr11:19693957 G>A) within the intron of the gene *SLC7A5*, one SNP (chr11: 19528555 C>T) in the UTR3 of the gene *ZCCHC14*, and three SNPs (chr11: 19656153 T>C and chr11:19656183 T>C) within the intron of the gene *KLHDC4*. These two genomic regions could be promising candidate regions for primary feather color trait in Longyan Shan-ma ducks.

### Function enrichment analysis results of 16 associated genes

For the 19 trait-associated genes that were identified, the top 10 gene ontology (GO) terms and Kyoto Encyclopedia of Genes and Genomes (KEGG) pathways are shown in Supplementary Tables S4, S5. Eight genes were significantly enriched in nucleus GO terms. They included *CLIC6*, *SIK1*, *LMO1*, *IPMK*, *CRYAA, SCGN*, *ZNF827*, and *TTBK2*. Seven genes were significantly enriched in cytoplasm GO terms, including *CLIC6*, *SIK1*, *IPMK*, *CRYAA*, *LOC101798015*, *TTBK2*, and *PHLPP1*. Five genes (*LOC101798015*, *IPMK*, *SLC7A5*, *CRYAA*, and *CAMK1G*) were enriched in one and more pathway. *LOC101798015* was enriched in four pathways, involving amino acids metabolism, including glycine, serine, and threonine metabolism, cysteine and methionine metabolism, biosynthesis of amino acids, and metabolic pathways. *IPMK* was enriched in three pathways, including inositol phosphate metabolism, phosphatidylinositol signaling system, and metabolic pathways, the first pathways involving in metabolism of inositol phosphate and phosphatidylinositol, respectively.

*STARD9* upregulates expression in black feather (TGTT) individual compared with in pockmarked (CCCC) individual.

**FIGURE 4:**
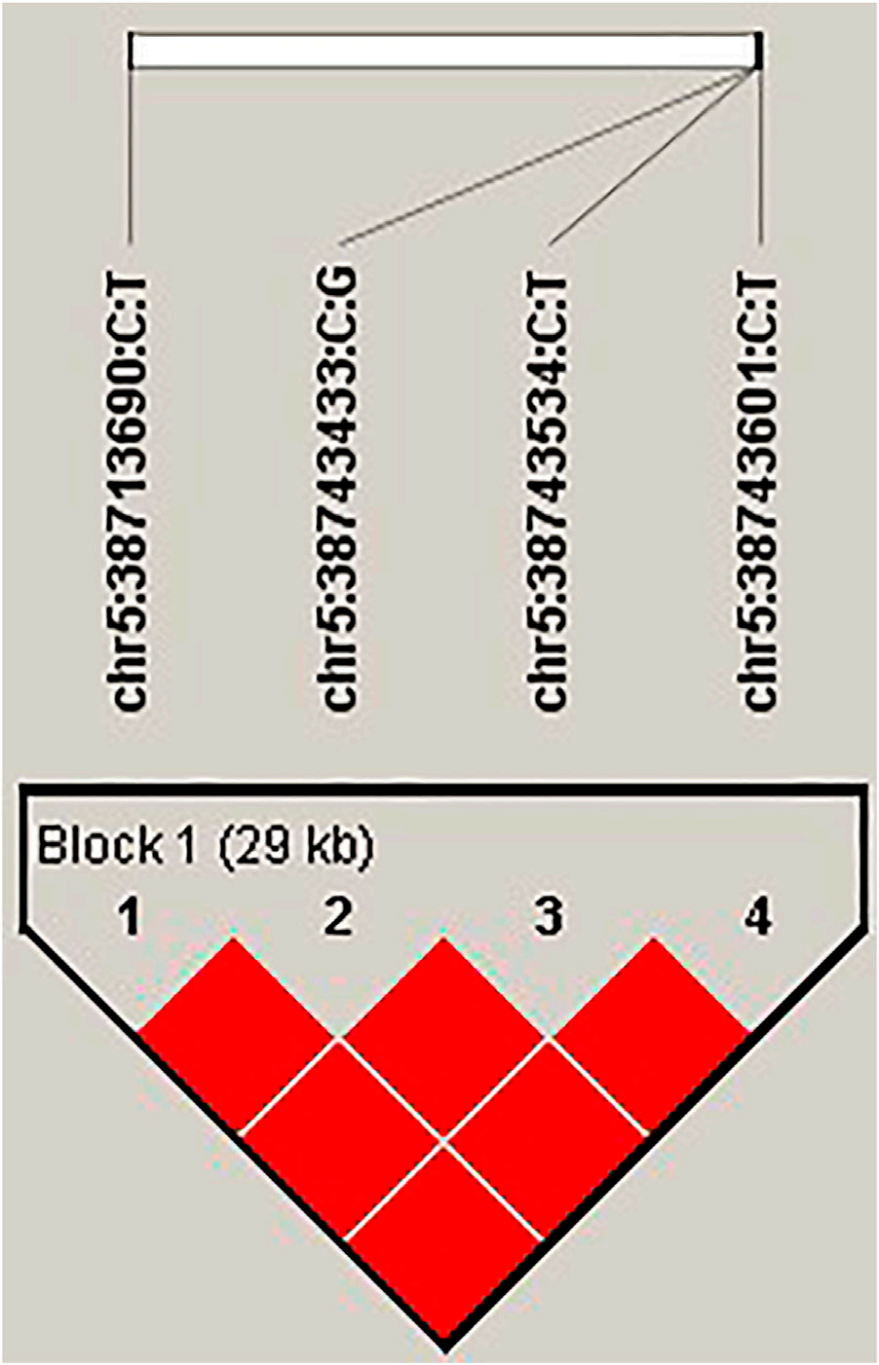
Four top SNPs within the intron of the gene *STARD9* within a haplotype block. Linkage disequilibrium and haplotype block of the gene *STARD9* was estimated using Haploview 4.1 software (Barrett et al., 2005) based on confidence intervals (Gabriel et al., 2002). Frequency of haplotype CCCC was 0.933. Frequency of haplotype TGTT was 0.067.

**FIGURE 5:**
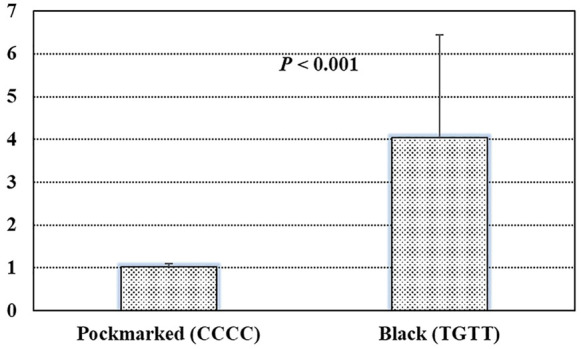
Results of real-time quantitative polymerase chain reaction in pockmarked (*n* = 3, haplotype-CCCC) and black (*n* = 3, haplotype-TGTT) feather bulb tissues.

Based on the GWAS results, four top SNPs (chr5:38713690 C>T, chr5:38743433 C>G, chr5:38743534 C>T, and chr5:38743601 C>T) within the intron of the gene *STARD9* were linked together as a 29-kb haplotype block (Figure 4). Therefore, the expression level of this gene was measured by real-time quantitative polymerase chain reaction (Q-PCR) in pockmarked (*n* = 3, haplotype-CCCC) and black (*n* = 3, haplotype-TGTT) feather bulb tissues. *STARD9* was significantly upregulated in tissues of black feather TGTT individual compared to pockmarked feather CCCC individual (4.04 ± 2.41 vs. 1.02 ± 0.08, *p* < 0.001) (Figure 5).

## Discussion

Birds have distinct and colorful pigmentation patterns (Chen et al., 2015). Pigmentation can affect the feather color of avian and pigmentation genes associated with color variation (Hubbard et al., 2010). In this study, a comprehensive GWSA was performed to identify candidate genes and regions for primary feather color trait in female Longyan Shan-ma ducks. We identified seven genome-wide significant SNPs within the introns of the genes *STARD9*, *ZNF106*, *SLC7A5*, and *BANP* genes, and two chromosome regions, including one 0.22 Mb region (38.65–38.87 Mb) on chromosome 5, and one 0.31 Mb region (19.53–19.84 Mb) on chromosome 11. Besides *ZNF106* and *SLC7A5*, 15 genome-wide suggestive associated genes were identified. Four top trait-associated SNPs were in the intron of the gene *STARD9*. The expression of this gene was upregulated in black feather (haplotype-CCCC) bulb tissue compared with in pockmarked feather (haplotype-TGTT) bulb tissue. The gene *STARD9* may be a key candidate gene for primary feather color trait in female Longyan Shan-ma ducks.

Of the 19 trait-associated genes identified, 13 genes are expressed in feather bulb tissues (Jiang et al., 2020). They included *STARD9*, *ZNF106*, *BANP*, *ZNF827*, *SCGN*, *TTBK2*, *TRIM66*, *ZCCHC14*, *PHLPP1*, *KLHDC4*, *IPMK*, *PLXNA2*, and *CAMK1G*. *STARD9* encodes StAR related lipid transfer domain containing 9. This protein promotes microtubule stability and regulates spindle microtubule dynamics (Srivastava and Panda, 2018). *STARD9* can drive primary feather follicle induction in poultry (Hu et al., 2020). Gene family members of the gene *STARD9*, such as *STAR1*, *STAR4* and *STAR5*, were associated with carotenoid coloration deposition in the carotenoid-containing feather of a wild bird (Walsh et al., 2012). Thus, *STARD9* is most likely linked to the development of feather bulbs and feather pigmentation. The present findings confirm the significant upregulated expression of the *STARD9* gene in black feather bulbs (haplotype-TGTT) compared to pockmarked bulbs (haplotype-CCCC). The haplotype-CCCC variant within the intron of *STARD9* could enhance the expression of this gene. Transcriptional regulatory elements in introns are ubiquitous (Majewski and Ott, 2002). This may be due to the presence of transcriptional regulatory elements in this intron of the *STARD9*. *STARD9* is implicated as a candidate gene for the primary feather color trait in Longyan Shan-ma ducks. *SLC7A5* encodes the solute carrier family 7 member 5 amino acid transporter. It can transport tyrosine, a key substrate for melanin synthesis (Wakamatsu and Ito, 2021). *SLC7A5* can regulate melanogenesis (Gaudel et al., 2020). The functions of the aforementioned genes on the development of feather bulbs and feather pigmentation require further study. *BANP* encodes BTG3 associated nuclear protein, an important tumor suppressor and cell cycle regulator, which can form a complex with p53 and negatively regulates p53 transcription (Grand et al., 2021). A previous study associated *BANP* with the genetics of fur color in the Arctic fox (Tietgen et al., 2021). *LOC101798015*, also known as *CBS*, encodes cystathionine beta synthase. This enzyme can affect feather follicle development in chickens (Humberto Vilar Da Silva et al., 2019; Vilar da Silva et al., 2020). As a pigment gene, *LOC101798015* is related with skin color in red tilapia (Zhu et al., 2016). *IPMK* encodes inositol polyphosphate multikinase, a member of the inositol phosphokinase superfamily. This protein is important in transcriptional and epigenetic regulation (Kim et al., 2016). In the present study, *IPMK* was enriched in the inositol phosphate metabolism and phosphatidylinositol signaling system pathways, which are related to the development of feather bulbs in ducks (Chen et al., 2017). For other identified genes, further studies are needed to determine the potential role in feather bulbs development and feather pigmentation.

Several limitations need to be considered in evaluating the present study. Because the number of black feathers in our population was relatively small, the simple classification of feather color trait into three categories according to the number of black-feather ducks may not reflect the genetic variation of feather color in the population of Shan-ma duck. Methods of feather color determination need to be further optimized. Since the pigment content of the feather bulbs determines feather color (Li et al., 2012), measuring the pigment content of the feathers may be a better indicator of feather color. Moreover, the formation of feather color is related to the genes that deposit melanin (Li et al., 2012; Guo et al., 2022). No genes associated with melanin deposition were detected, future studies will need to add more samples to improve statistical power. However, the strength of the present study is that the roles of several genes (*STARD9*, *SLC7A*5, *BANP*, *LOC101798015*, and *IPMK*) in feather pigmentation in duck were first confirmed, especially *STARD9*.

## Conclusion

A comprehensive GWAS identified 19 promising candidate genes and two possible candidate regions for the primary feather color trait in Longyan Shan-ma ducks. Five trait-associated genes related to the development of feather bulbs and feather pigmentation were identified (*STARD9*, *SLC7A*5, *BANP*, *LOC101798015*, and *IPMK*). These genes, especially *STARD9*, is an important candidate gene for the primary feather trait. This study provides a theoretical basis for further studies of the genetic molecular mechanism of feather color trait in ducks, and marker-assisted selection breeding program of Longyan Shan-ma ducks.

## Data Availability

The datasets presented in this study can be found in online repositories. The names of the repository/repositories and accession number (s) can be found below: http://bigd.big.ac.cn/gvm/getProjectDetail?project=GVM000369, GVM000369.
