# Supplementary material for: Genome-wide association study for the primary feather color trait in a native Chinese duck
Source: Front Genet. 2023 Mar 3;14:1065033. doi: 10.3389/fgene.2023.1065033 (PMC10020179; doi:10.3389/fgene.2023.1065033)
Supplement: Supplementary file 1 [file DataSheet1.pdf]

Supplementary Material

Supplementary Table 1. Basic information for SNP markers on a physical map.

Chromosome	Distance (Mb)[1]	No. of SNPs	SNP/Mb	No. of independent SNPs	Independent SNPs/Mb
1	202.84	9,911	48.86	1,507	7.43
2	155.79	7,488	48.06	1,217	7.81
3	117.74	6,116	51.94	966	8.20
4	76.13	4,026	52.88	619	8.13
5	64.37	4,252	66.06	703	10.92
6	33.43	2,311	69.13	428	12.80
7	39.2	2,120	54.08	336	8.57
8	36.69	2,479	67.57	385	10.49
9	26.28	2,163	82.31	394	14.99
10	19.69	1,242	63.08	202	10.26
11	21.89	2,177	99.45	354	16.17
12	21.28	1,028	48.31	143	6.72
13	22.09	2,066	93.53	368	16.66
14	18.84	1,607	85.30	292	15.50
15	17.86	1,545	86.51	234	13.10
16	12.63	1,132	89.63	179	14.17

17	1.32	30	22.73	10	7.58
18	11.76	1,420	120.75	219	18.62
19	10.04	989	98.51	146	14.54
20	12.5	1,546	123.68	246	19.68
21	15.92	1,382	86.81	231	14.51
22	8.29	1,072	129.31	169	20.39
23	2.32	278	119.83	62	26.72
24	6.5	687	105.69	128	19.69
25	7.49	974	130.04	179	23.90
26	1.43	134	93.71	47	32.87
27	5.82	819	140.72	194	33.33
28	7.19	766	106.54	169	23.50
29	4.59	506	110.24	83	18.08
Total	981.92	62,266	63.41	10,210	10.40

[1]The physical length of the chromosome based on duck reference genome PK-2015 (https://www.ncbi.nlm.nih.gov/genome/2793?genome_assembly_id=426073).

Supplementary Table 2. Result of principal component significance test[1].

Principal component	Eigenvalue	Difference	Twstat	P value	Effect
1	16.57	NA	0.08	0.15	90.25
2	12.49	-4.08	NA	NA	NA
3	11.40	-1.09	NA	NA	NA
4	10.55	-0.85	NA	NA	NA
5	9.08	-1.47	NA	NA	NA
6	8.82	-0.26	NA	NA	NA
7	7.74	-1.09	NA	NA	NA
8	7.27	-0.46	NA	NA	NA
9	5.40	-1.87	NA	NA	NA
10	4.26	-1.14	NA	NA	NA

Note: [1]Principal component significance test was conducted using EIGENSTRAT version 6.1.4 package (https://github.com/DReichLab/EIG).

Supplementary Table 3. SNPs with genome-wide suggestive significance associations for plumage color trait.

Chr	SNP	BP (PK-2015)[1]	A1/A2	A1_MAF[2]	OR (95% CI)[3]	*P*-value	Gene	Feature[4]
1	chr1:80265154 C>A	80,265,154	A/C	0.081	1.35 (1.19 ± 1.53)	6.00×10^{-6}	*LOC101798015*	UTR5
1	chr1:80401171 G>A	80,401,171	A/G	0.059	1.43 (1.24 ± 1.64)	1.61×10^{-6}	*CRYAA*, *SIK1*	intergenic
1	chr1:80415698 G>A	80,415,698	A/G	0.055	1.41 (1.22 ± 1.64)	6.41×10^{-6}	*CRYAA*, *SIK1*	intergenic
1	chr1:93588263 G>A	93,588,263	G/A	0.059	1.34 (1.18 ± 1.53)	1.49×10^{-5}	*CLIC6*	intron
2	chr2:77662136 C>T	77,662,136	T/C	0.090	1.31 (1.17 ± 1.47)	8.07×10^{-6}	*PHLPP1*	intron
2	chr2:77662182 G>A	77,662,182	A/G	0.090	1.31 (1.17 ± 1.47)	8.07×10^{-6}	*PHLPP1*	intron
2	chr2:94385810 C>T	94,385,810	T/C	0.162	1.24 (1.13 ± 1.35)	2.86×10^{-6}	*SCGN*	exon5 c.C338T, p.S113N
4	chr4:34697042 A>G	34,697,042	G/A	0.145	1.24 (1.14 ± 1.35)	8.80×10^{-7}	*ZNF827*	intron
5	chr5:38647398 G>T	38,647,398	T/G	0.107	1.34 (1.19 ± 1.51)	2.10×10^{-6}	*ZNF106*	intron
5	chr5:38647483 A>G	38,647,483	G/A	0.107	1.34 (1.19 ± 1.51)	2.10×10^{-6}	*ZNF106*	intron
5	chr5:38866077 T>C	38,866,077	C/T	0.064	1.42 (1.23 ± 1.64)	3.08×10^{-6}	*TTBK2*	intron

6	chr6:31974889 C>A	31,974,889	A/C	0.160	1.25 (1.13 ± 1.38)	1.09×10^{-5}	*IPMK*	intron
6	chr6:31974946 G>A	31,974,946	A/G	0.160	1.25 (1.13 ± 1.38)	1.09×10^{-5}	*IPMK*	intron
7	chr7:1745769 G>T	1,745,769	T/G	0.088	1.30 (1.16 ± 1.47)	1.58×10^{-5}	*LMO1*	intron
7	chr7:1745776 A>G	1,745,776	G/A	0.088	1.30 (1.16 ± 1.47)	1.58×10^{-5}	*LMO1*	intron
7	chr7:1941933 C>T	1,941,933	T/C	0.243	1.20 (1.11 ± 1.29)	4.25×10^{-6}	*LMO1*, *TRIM66*	intergenic
7	chr7:1955797 A>G	1,955,797	G/A	0.241	1.20 (1.11 ± 1.29)	3.66×10^{-6}	*LMO1*, *TRIM66*	intergenic
11	chr11:19528555 C>T	19,528,555	T/C	0.105	1.31 (1.17 ± 1.47)	4.23×10^{-6}	*ZCCHC14*	UTR3
11	chr11:19656153 T>C	19,656,153	C/T	0.147	1.26 (1.14 ± 1.39)	9.76×10^{-6}	*KLHDC4*	intron
11	chr11:19656183 T>C	19,656,183	T/C	0.152	1.25 (1.13 ± 1.38)	1.56×10^{-5}	*KLHDC4*	intron
11	chr11:19695701 G>A	19,695,701	A/G	0.097	1.32 (1.18 ± 1.48)	2.01×10^{-6}	*SLC7A5*	intron
27	chr27:1372970 C>A	1,372,970	C/A	0.071	1.31 (1.16 ± 1.49)	1.53×10^{-5}	*PLXNA2*, *CAMK1G*	intergenic

Note: [1]BP, physical position based on duck reference genome PK-2015 (https://www.ncbi.nlm.nih.gov/genome/2793?genome_assembly_id=426073); [2]MAF, minor allele frequency; [3]OR, odds ratio; 95% CI, 95% confidence interval; [4]UTR5, 5' untranslated region; UTR3, 3' untranslated region.

Supplementary Table 4. Results of GO enrichment analysis for identified associated genes.

GO term	ID	*P*-value	FDR *P*-value[1]	Gene
Nucleus	GO:0005634	3.62×10^{-4}	3.67×10^{-2}	*CLIC6*, *SIK1*, *LMO1*, *IPMK*, *CRYAA*, *SCGN*, *ZNF827*, *TTBK2*
Protein serine/threonine kinase activity	GO:0004674	5.88×10^{-4}	3.67×10^{-2}	*TTBK2*, *SIK1*, *CAMK1G*
Regulation of cell migration	GO:0030334	1.07×10^{-3}	4.33×10^{-2}	*TTBK2*, *PLXNA2*
Cytoplasm	GO:0005737	1.38×10^{-3}	4.33×10^{-2}	*CLIC6*, *SIK1*, *IPMK*, *CRYAA*, *LOC101798015*, *TTBK2*, *PHLPP1*
Microtubule cytoskeleton organization	GO:0000226	2.21×10^{-3}	4.51×10^{-2}	*TTBK2*, *SIK1*
Peptidyl-serine phosphorylation	GO:0018105	3.86×10^{-3}	4.51×10^{-2}	*TTBK2*, *CAMK1G*
Semaphorin receptor complex	GO:0002116	4.77×10^{-3}	4.51×10^{-2}	*PLXNA2*
Entrainment of circadian clock	GO:0009649	4.77×10^{-3}	4.51×10^{-2}	*PHLPP1*
Anoikis	GO:0043276	5.56×10^{-3}	4.51×10^{-2}	*SIK1*
Transsulfuration	GO:0019346	5.56×10^{-3}	4.51×10^{-2}	*LOC101798015*

[1]FDR, false discovery rate, using Benjamini and Hochberg method.

Supplementary Table 5. Results of pathway enrichment analysis for identified associated genes.

Gene	Pathway
LOC101798015	Glycine, serine and threonine metabolism; Cysteine and methionine metabolism; Biosynthesis of amino acids; and Metabolic pathways
IPMK	Inositol phosphate metabolism; Phosphatidylinositol signaling system; Metabolic pathways
SLC7A5	mTOR signaling pathway
CRYAA	Protein processing in endoplasmic reticulum
CAMK1G	Calcium signaling pathway

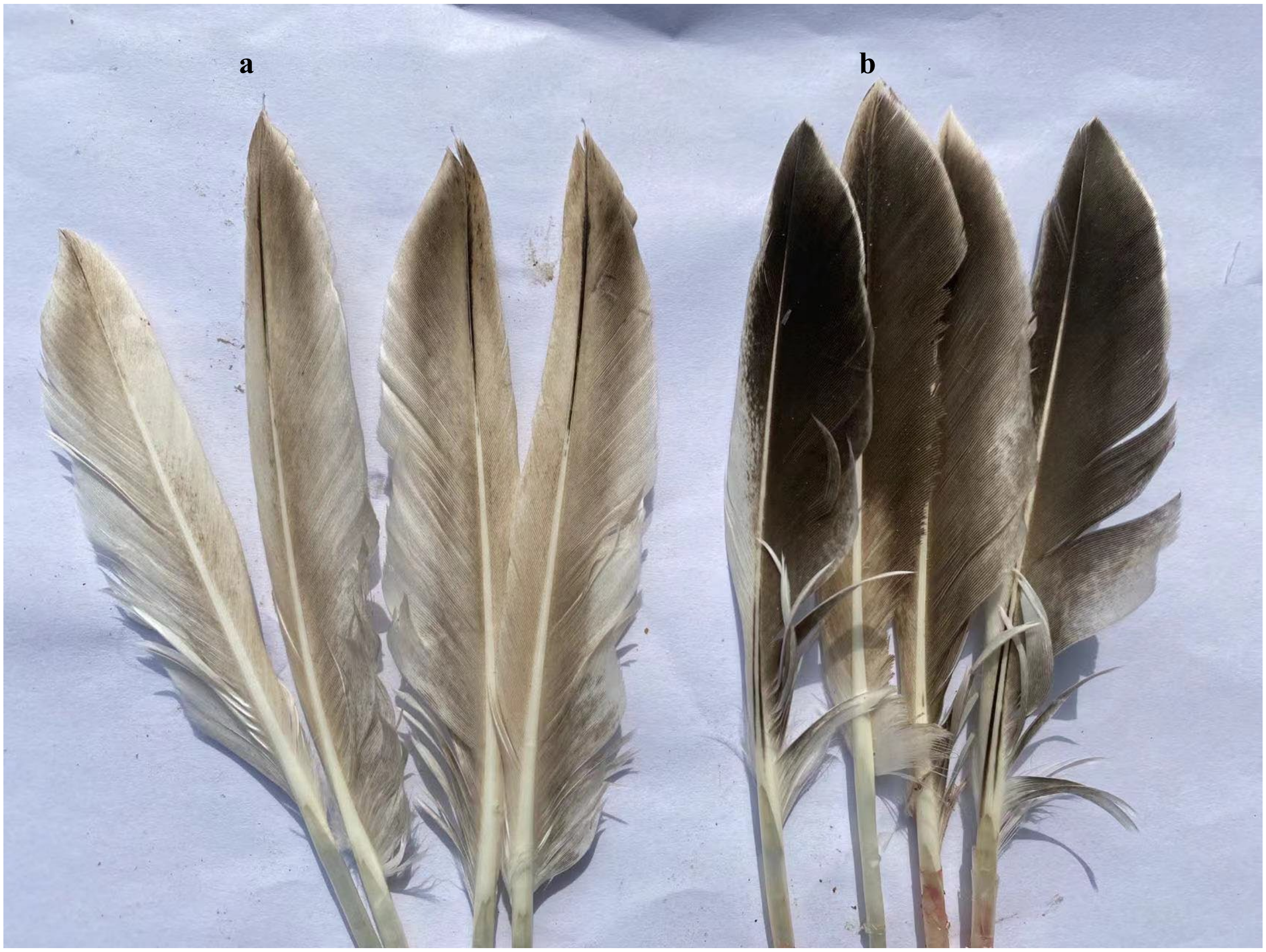

Supplementary Figure 1. Pockmarked (a) and black (b) feathers traits of Longyan Shan-ma duck.
